# Supplementary material for: Socioeconomic position, social mobility, and health selection effects on allostatic load in the United States
Source: PLoS One. 2021 Aug 4;16(8):e0254414. doi: 10.1371/journal.pone.0254414 (PMC8336836; doi:10.1371/journal.pone.0254414)
Supplement: S1 Table — Note: Number of observations—4,713. (DOCX) [file pone.0254414.s001.docx]

| Variable | Mean | SD | Min | Max |
| --- | --- | --- | --- | --- |
| (1) Lipid (Total to High-Density Lipoprotein Cholesterol) | 5,45 | 2,88 | 1,00 | 10,00 |
| (2) Glucose (Glucose MG/DL) | 107,30 | 29,34 | 40,00 | 505,00 |
| (3) Inflammation (C-reactive protein (CRP)) | 5,13 | 9,13 | 0,08 | 205,01 |
| (4) Body Mass Index (BMI) | 29,26 | 7,39 | 15,40 | 70,30 |
| (5a) Cardiovascular (systolic blood pressure) | 124,42 | 13,80 | 86,50 | 212,00 |
| (5b) Cardiovascular (diastolic blood pressure) | 79,06 | 10,36 | 49,00 | 145,00 |
| (5c) Cardiovascular (resting heart rate) | 74,58 | 12,13 | 42,00 | 196,00 |
