# Supplementary material for: Socioeconomic position, social mobility, and health selection effects on allostatic load in the United States
Source: PLoS One. 2021 Aug 4;16(8):e0254414. doi: 10.1371/journal.pone.0254414 (PMC8336836; doi:10.1371/journal.pone.0254414)
Supplement: S2 Table — Note: Number of observations—4,713. (DOCX) [file pone.0254414.s002.docx]

|  | AL | BMI Wave I | Worse than very good self-rated health at Wave I | Chronic health Wave I | CESD  Wave I |
| --- | --- | --- | --- | --- | --- |
| BMI Wave I | 0.43 | 1.00 |  |  |  |
| Worse than very good self-rated health at Wave I | 0.12 | 0.20 | 1.00 |  |  |
| Chronic health Wave I | 0.03 | 0.03 | 0.05 | 1.00 |  |
| CESD Wave I | 0.02 | 0.05 | 0.10 | 0.04 | 1.00 |
