# Supplementary material for: Socioeconomic position, social mobility, and health selection effects on allostatic load in the United States
Source: PLoS One. 2021 Aug 4;16(8):e0254414. doi: 10.1371/journal.pone.0254414 (PMC8336836; doi:10.1371/journal.pone.0254414)
Supplement: S3 Table — Notes: * p < 0.05, ** p < 0.01, *** p < 0.001, 95% confidence intervals in parentheses. (DOCX) [file pone.0254414.s003.docx]

|  | Model 1 | Model 2 | Model 3 | Model 4 | Model 5 | Model 6 |
| --- | --- | --- | --- | --- | --- | --- |
| *Initial health at Wave I* |  |  |  |  |  |  |
| Poor self-rated health | 0.29^***^ | –––– | –––– | –––– | –––– | 0.08^**^ |
|  | [0.23,0.35] | –––– | –––– | –––– | –––– | [0.02,0.14] |
| Poor health rated by parents | –––– | 0.22^***^ | –––– | –––– | –––– | 0.07^*^ |
|  | –––– | [0.15,0.29] | –––– | –––– | –––– | [0.01,0.13] |
| CES-D scale of depressive symptoms | –––– | –––– | 0.01^**^ | –––– | –––– | 0.01 |
|  | –––– | –––– | [0.00,0.02] | –––– | –––– | [-0.00,0.02] |
| BMI Wave I | –––– | –––– | –––– | 0.09^***^ | –––– | 0.09^***^ |
|  | –––– | –––– | –––– | [0.09,0.10] | –––– | [0.08,0.09] |
| Chronic health Wave I | –––– | –––– | –––– | –––– | 0.19^*^ | 0.08 |
|  | –––– | –––– | –––– | –––– | [0.00,0.38] | [-0.10,0.26] |
| *Socio-demographic controls* |  |  |  |  |  |  |
| Age | 0.05^***^ | 0.05^***^ | 0.05^***^ | 0.01 | 0.05^***^ | 0.01 |
|  | [0.03,0.06] | [0.03,0.06] | [0.03,0.06] | [-0.01,0.02] | [0.03,0.06] | [-0.01,0.02] |
| Male | 0.35^***^ | 0.33^***^ | 0.33^***^ | 0.32^***^ | 0.32^***^ | 0.33^***^ |
|  | [0.29,0.40] | [0.28,0.39] | [0.27,0.38] | [0.27,0.36] | [0.26,0.37] | [0.28,0.38] |
| *Race/ethnicity (ref. white)* |  |  |  |  |  |  |
| Black | 0.20^***^ | 0.14^***^ | 0.20^***^ | 0.07^*^ | 0.20^***^ | 0.04 |
|  | [0.14,0.27] | [0.07,0.22] | [0.13,0.26] | [0.00,0.13] | [0.14,0.27] | [-0.02,0.11] |
| Hispanic | 0.10^*^ | 0.09 | 0.12^**^ | 0.06 | 0.12^**^ | 0.04 |
|  | [0.01,0.19] | [-0.01,0.18] | [0.03,0.22] | [-0.02,0.14] | [0.03,0.21] | [-0.05,0.13] |
| Other | -0.00 | -0.03 | 0.03 | 0.03 | 0.04 | -0.04 |
|  | [-0.14,0.14] | [-0.18,0.12] | [-0.11,0.16] | [-0.10,0.15] | [-0.10,0.17] | [-0.18,0.10] |
| Married (ref. unmarried) | 0.01 | 0.00 | -0.00 | 0.00 | -0.00 | 0.01 |
|  | [-0.05,0.07] | [-0.06,0.06] | [-0.06,0.05] | [-0.05,0.05] | [-0.06,0.06] | [-0.05,0.06] |
| Rural | 0.11^***^ | 0.09^**^ | 0.11^***^ | 0.06^*^ | 0.11^***^ | 0.05 |
|  | [0.05,0.17] | [0.02,0.15] | [0.05,0.17] | [0.01,0.12] | [0.05,0.17] | [-0.00,0.11] |
| AIC | 13671.36 | 12098.67 | 13734.51 | 12307.91 | 13762.90 | 10848.84 |
| BIC | 13729.92 | 12156.11 | 13793.05 | 12366.22 | 13821.46 | 10931.44 |
| Observations | 4713 | 4713 | 4713 | 4713 | 4713 | 4713 |
