# Supplementary material for: Socioeconomic position, social mobility, and health selection effects on allostatic load in the United States
Source: PLoS One. 2021 Aug 4;16(8):e0254414. doi: 10.1371/journal.pone.0254414 (PMC8336836; doi:10.1371/journal.pone.0254414)
Supplement: S4 Table — Notes: * p < 0.05, ** p < 0.01, *** p < 0.001, 95% confidence intervals in parentheses. (DOCX) [file pone.0254414.s004.docx]

|  | Model 1 | Model 2 |
| --- | --- | --- |
| *Immobile socioeconomic quintiles* |  |  |
| Lowest | 0.16^***^ | 0.19^***^ |
|  | [0.09,0.23] | [0.12,0.26] |
| Middle-low | 0.08 | 0.06 |
|  | [-0.01,0.16] | [-0.03,0.14] |
| Middle | 0.11^**^ | 0.11^**^ |
|  | [0.04,0.19] | [0.03,0.18] |
| Middle-high | -0.09^*^ | -0.10^*^ |
|  | [-0.17,-0.01] | [-0.19,-0.02] |
| Highest | -0.26^***^ | -0.25^***^ |
|  | [-0.34,-0.18] | [-0.32,-0.17] |
| *Weight parameters* |  |  |
| Origin | 0.55^***^ | 0.44^**^ |
|  | [0.34,0.77] | [0.18,0.71] |
| Destination | 0.45^***^ | 0.56^***^ |
|  | [0.23,0.66] | [0.29,0.83] |
| *Social mobility* |  |  |
| Short-range upward | -0.11^*^ | –––– |
|  | [-0.20,-0.02] | –––– |
| Long-range upward | -0.07 | –––– |
|  | [-0.18,0.03] | –––– |
| Short-range downward | –––– | 0.06 |
|  | –––– | [-0.03,0.16] |
| Long-range downward | –––– | 0.01 |
|  | –––– | [-0.11,0.12] |
| *Socio-demographic controls* |  |  |
| Age | 0.05^***^ | 0.05^***^ |
|  | [0.04,0.07] | [0.04,0.07] |
| Male | 0.31^***^ | 0.310^***^ |
|  | [0.25,0.37] | [0.25,0.37] |
| *Race/ethnicity (ref. white)* |  |  |
| Black | 0.18^***^ | 0.18^***^ |
|  | [0.11,0.25] | [0.11,0.25] |
| Hispanic | 0.07 | 0.07 |
|  | [-0.02,0.17] | [-0.03,0.17] |
| Other | 0.07 | 0.07 |
|  | [-0.07,0.21] | [-0.07,0.21] |
| Married (ref. unmarried) | 0.001 | -0.0002 |
|  | [-0.06,0.06] | [-0.06,0.06] |
| Rural | 0.08* | 0.08* |
|  | [0.02,0.14] | [0.02,0.14] |
| AIC | 12719.22 | 12722.92 |
| BIC | 12822.17 | 12825.87 |
| *Observations* | 4713 | 4713 |
