# Supplementary material for: Socioeconomic position, social mobility, and health selection effects on allostatic load in the United States
Source: PLoS One. 2021 Aug 4;16(8):e0254414. doi: 10.1371/journal.pone.0254414 (PMC8336836; doi:10.1371/journal.pone.0254414)
Supplement: S5 Table — Notes: * p < 0.05, ** p < 0.01, *** p < 0.001, 95% confidence intervals in parentheses. Neighbourhood controls capture the most localized available contextual information (approx. 452 housing units, block groups) of the areas in which individuals lived at Wave IV. For modal neighbourhood race categories include white (ref.), black, other; poverty measure represents the proportion of persons with income below poverty level. Based on the distribution of proportion of persons below poverty level in 1989. Low = neighbourhoods where the proportion of the population with income below poverty level was less than 11.6%, the median proportion; medium = between 11.6% and 23.9%; and high (ref.) = above 23.9%; for unemployment rate: Low = neighbourhoods with an unemployment rate less than 6.5%, the median rate, medium = between 6.5% and 10.9%; high = 10.9% or higher. (DOCX) [file pone.0254414.s005.docx]

|  | Model 1 | Model 2 | Model 3 |
| --- | --- | --- | --- |
| *Immobile socioeconomic quintiles* |  |  |  |
| Lowest | 0.16^***^ | 0.13^***^ | 0.11^**^ |
|  | [0.09,0.24] | [0.06,0.20] | [0.04,0.18] |
| Middle-low | 0.06 | 0.06 | 0.05 |
|  | [-0.02,0.15] | [-0.03,0.15] | [-0.03,0.14] |
| Middle | 0.11^**^ | 0.11^**^ | 0.11^**^ |
|  | [0.04,0.19] | [0.04,0.19] | [0.03,0.18] |
| Middle-high | -0.10^*^ | -0.08^*^ | -0.08 |
|  | [-0.18,-0.02] | [-0.17,-0.00] | [-0.16,0.00] |
| Highest | -0.24^***^ | -0.22^***^ | -0.19^***^ |
|  | [-0.32,-0.16] | [-0.30,-0.14] | [-0.27,-0.11] |
| *Weight parameters* |  |  |  |
| Origin | 0.56^***^ | 0.56^***^ | 0.55^**^ |
|  | [0.25,0.86] | [0.25,0.87] | [0.22,0.88] |
| Destination | 0.44^**^ | 0.44^**^ | 0.45^**^ |
|  | [0.14,0.75] | [0.13,0.75] | [0.12,0.78] |
| *Social mobility* |  |  |  |
| Short-range upward | -0.10^*^ | -0.10^*^ | -0.10^*^ |
|  | [-0.19,-0.01] | [-0.19,-0.01] | [-0.19,-0.01] |
| Long-range upward | -0.07 | -0.08 | -0.08 |
|  | [-0.18,0.04] | [-0.18,0.02] | [-0.18,0.02] |
| Short-range downward | 0.04 | 0.04 | 0.03 |
|  | [-0.06,0.14] | [-0.06,0.13] | [-0.06,0.13] |
| Long-range downward | -0.00 | 0.00 | -0.00 |
|  | [-0.11,0.11] | [-0.10,0.11] | [-0.10,0.10] |
| *Socio-demographic controls* |  |  |  |
| Age | 0.05^***^ | 0.05^***^ | 0.05^***^ |
|  | [0.04,0.07] | [0.04,0.07] | [0.04,0.07] |
| Male | 0.31^***^ | 0.31^***^ | 0.31^***^ |
|  | [0.25,0.37] | [0.25,0.37] | [0.26,0.37] |
| *Race/ethnicity (ref. white)* |  |  |  |
| Black | 0.22^***^ | 0.19^***^ | 0.18^***^ |
|  | [0.13,0.31] | [0.10,0.28] | [0.09,0.27] |
| Hispanic | 0.08 | 0.04 | 0.04 |
|  | [-0.02,0.18] | [-0.06,0.14] | [-0.06,0.14] |
| Other | 0.08 | 0.08 | 0.08 |
|  | [-0.07,0.23] | [-0.07,0.23] | [-0.07,0.23] |
| Married (ref. unmarried) | -0.01 | -0.01 | -0.02 |
|  | [-0.06,0.05] | [-0.07,0.05] | [-0.07,0.04] |
| Rural | 0.08^*^ | 0.04 | 0.03 |
|  | [0.02,0.14] | [-0.02,0.11] | [-0.03,0.10] |
| *Neighbourhood controls:* |  |  |  |
| Black (ref. White) | -0.06 | -0.13^*^ | -0.17^**^ |
|  | [-0.17,0.04] | [-0.24,-0.03] | [-0.27,-0.06] |
| Other race | -0.02 | -0.05 | -0.07 |
|  | [-0.19,0.16] | [-0.22,0.13] | [-0.25,0.11] |
| Low poverty (ref. high) | –––– | -0.19^***^ | -0.14^***^ |
|  | –––– | [-0.28,-0.11] | [-0.22,-0.05] |
| Medium poverty | –––– | -0.06 | -0.03 |
|  | –––– | [-0.15,0.03] | [-0.11,-0.06] |
| Medium unemployment (ref. low) | –––– | –––– | 0.08^*^ |
|  | –––– | –––– | [0.01,0.15] |
| High unemployment | –––– | –––– | 0.14^**^ |
|  | –––– | –––– | [0.05,0.23] |
| AIC | 12724.73 | 12701.74 | 12693.50 |
| BIC | 12853.42 | 12843.29 | 12847.93 |
| Observations | 4713 | 4713 | 4713 |
|  | | | |
