# Supplementary material for: Socioeconomic position, social mobility, and health selection effects on allostatic load in the United States
Source: PLoS One. 2021 Aug 4;16(8):e0254414. doi: 10.1371/journal.pone.0254414 (PMC8336836; doi:10.1371/journal.pone.0254414)
Supplement: S6 Table — Notes: * p < 0.05, ** p < 0.01, *** p < 0.001, 95% confidence intervals in parentheses. (DOCX) [file pone.0254414.s006.docx]

|  | Model 1 | Model 2 | Model 3 | Model 4 | Model 5 |
| --- | --- | --- | --- | --- | --- |
|  | Mobility (-5 to 5 with OLS) | Mobility(-5 to 5 with OLS) | Mobility (-5 to 5 with ordered logit) | Upward (with Probit) | Downward (with Probit) |
| *Social origins* |  |  |  |  |  |
| Parental occupation | –––– | 0.56^***^ | 0.73^***^ | 0.17^***^ | -0.17^***^ |
|  | –––– | [0.53,0.59] | [0.69,0.78] | [0.14,0.21] | [-0.20,-0.13] |
| Parental education | –––– | -0.26^***^ | -0.31^***^ | -0.04 | 0.04^*^ |
|  | –––– | [-0.30,-0.23] | [-0.35,-0.26] | [-0.07,0.00] | [0.01,0.08] |
| *Socio-demographic controls* |  |  |  |  |  |
| Age | 0.02 | 0.01 | 0.02 | 0.00 | -0.01 |
|  | [-0.01,0.04] | [-0.01,0.04] | [-0.01,0.05] | [-0.02,0.03] | [-0.03,0.02] |
| Male | -0.31^***^ | -0.28^***^ | -0.39^***^ | -0.06 | 0.09^*^ |
|  | [-0.40,-0.21] | [-0.36,-0.21] | [-0.50,-0.29] | [-0.15,0.03] | [0.01,0.18] |
| *Race/ethnicity (ref. white)* |  |  |  |  |  |
| Black | -0.19^**^ | -0.20^***^ | -0.32^***^ | -0.18^**^ | 0.10 |
|  | [-0.30,-0.08] | [-0.30,-0.11] | [-0.45,-0.19] | [-0.29,-0.06] | [-0.01,0.20] |
| Hispanic | 0.59^***^ | 0.05 | 0.08 | -0.07 | -0.08 |
|  | [0.44,0.75] | [-0.08,0.18] | [-0.10,0.26] | [-0.22,0.08] | [-0.23,0.08] |
| Other | 0.20 | 0.21^*^ | 0.25 | 0.03 | 0.06 |
|  | [-0.04,0.43] | [0.01,0.40] | [-0.02,0.52] | [-0.19,0.25] | [-0.15,0.28] |
| Married (ref. unmarried) | 0.26^***^ | 0.22^***^ | 0.33^***^ | 0.10^*^ | -0.07 |
|  | [0.16,0.36] | [0.14,0.30] | [0.22,0.44] | [0.01,0.20] | [-0.16,0.01] |
| Rural | 0.09 | -0.14^**^ | -0.23^***^ | -0.06 | 0.08 |
|  | [-0.01,0.19] | [-0.22,-0.05] | [-0.35,-0.11] | [-0.16,0.04] | [-0.01,0.18] |
| Poor initial health at Wave I | -0.20^***^ | -0.35^***^ | -0.51^***^ | -0.23^***^ | 0.13^**^ |
|  | [-0.30,-0.10] | [-0.43,-0.27] | [-0.62,-0.39] | [-0.32,-0.13] | [0.03,0.22] |
| Cutting points 1 | –––– | –––– | -3.37^***^ | –––– | –––– |
|  | –––– | –––– | [-4.30,-2.45] | –––– | –––– |
| Cutting points 2 | –––– | –––– | -1.66^***^ | –––– | –––– |
|  | –––– | –––– | [-2.56,-0.76] | –––– | –––– |
| Cutting points 3 | –––– | –––– | -0.32 | –––– | –––– |
|  | –––– | –––– | [-1.22,0.57] | –––– | –––– |
| Cutting points 4 | –––– | –––– | 0.87 | –––– | –––– |
|  | –––– | –––– | [-0.02,1.77] | –––– | –––– |
| Cutting points 5 | –––– | –––– | 2.44^***^ | –––– | –––– |
|  | –––– | –––– | [1.53,3.34] | –––– | –––– |
| Cutting points 6 | –––– | –––– | 3.58^***^ | –––– | –––– |
|  | –––– | –––– | [2.68,4.48] | –––– | –––– |
| Cutting points 7 | –––– | –––– | 4.90^***^ | –––– | –––– |
|  | –––– | –––– | [3.99,5.81] | –––– | –––– |
| Cutting points 8 | –––– | –––– | 6.17^***^ | –––– | –––– |
|  | –––– | –––– | [5.24,7.10] | –––– | –––– |
| AIC | 17406.21 | 15575.23 | 15624.77 | 4028.29 | 4377.08 |
| BIC | 17464.16 | 15646.04 | 15740.65 | 4099.11 | 4447.89 |
| Observations | 4716 | 4716 | 4716 | 4716 | 4716 |
