# Supplementary material for: Socioeconomic position, social mobility, and health selection effects on allostatic load in the United States
Source: PLoS One. 2021 Aug 4;16(8):e0254414. doi: 10.1371/journal.pone.0254414 (PMC8336836; doi:10.1371/journal.pone.0254414)
Supplement: S7 Table — Notes: * p < 0.05, ** p < 0.01, *** p < 0.001. 95% confidence intervals in parentheses. (DOCX) [file pone.0254414.s007.docx]

|  | 1 step upward | 2-4 steps upward | 1 step downward | 2-4 steps downward |
| --- | --- | --- | --- | --- |
| *Weight parameters* |  |  |  |  |
| Origin | 0.57^***^ | 0.57^***^ | 0.57^***^ | 0.58^***^ |
|  | [0.24,0.89] | [0.25,0.90] | [0.25,0.90] | [0.26,0.90] |
| Destination | 0.43^**^ | 0.43^**^ | 0.43^**^ | 0.42^*^ |
|  | [0.11,0.76] | [0.10,0.75] | [0.10,0.75] | [0.10,0.74] |
| *Social mobility* |  |  |  |  |
| Short-range upward | -0.04 | -0.08 | -0.08 | -0.08 |
|  | [-0.14,0.06] | [-0.17,0.01] | [-0.17,0.01] | [-0.17,0.01] |
| Long-range upward | -0.05 | -0.06 | -0.06 | -0.06 |
|  | [-0.16,0.05] | [-0.17,0.06] | [-0.16,0.05] | [-0.16,0.05] |
| Short-range downward | 0.03 | 0.04 | 0.01 | 0.04 |
|  | [-0.06,0.13] | [-0.06,0.13] | [-0.10,0.12] | [-0.06,0.13] |
| Long-range downward | -0.01 | -0.01 | -0.01 | 0.01 |
|  | [-0.12,0.10] | [-0.11,0.10] | [-0.11,0.10] | [-0.11,0.13] |
| *Mobility * poor health at Wave I* | -0.17^*^ | 0.00 | 0.08 | -0.05 |
|  | [-0.34,0.002] | [-0.17,0.17] | [-0.07,0.23] | [-0.20,0.10] |
| *Poor health at Wave I* | 0.27^***^ | 0.25^***^ | 0.23^***^ | 0.26^***^ |
|  | [0.21,0.34] | [0.18,0.32] | [0.17,0.30] | [0.19,0.33] |
| Chronic health condition Wave I | 0.13 | 0.13 | 0.13 | 0.13 |
|  | [-0.07,0.33] | [-0.06,0.33] | [-0.06,0.33] | [-0.06,0.33] |
| Observations | 4714 | 4714 | 4714 | 4714 |
| AIC | 12663.61 | 12667.53 | 12666.39 | 12667.11 |
| BIC | 12792.30 | 12796.22 | 12795.08 | 12795.80 |
