# Supplementary material for: Socioeconomic position, social mobility, and health selection effects on allostatic load in the United States
Source: PLoS One. 2021 Aug 4;16(8):e0254414. doi: 10.1371/journal.pone.0254414 (PMC8336836; doi:10.1371/journal.pone.0254414)
Supplement: S8 Table — Notes: * p < 0.05, ** p < 0.01, *** p < 0.001, 95% confidence intervals in parentheses. (DOCX) [file pone.0254414.s008.docx]

|  | Model 1 | Model 2 | Model 3 | Model 4 | Model 5 |
| --- | --- | --- | --- | --- | --- |
| Immobile educational quintiles |  |  |  |  |  |
| Lowest | 0.14^**^ | 0.14^**^ | 0.12^*^ | 0.16^**^ | 0.13^*^ |
|  | [0.04,0.24] | [0.04,0.24] | [0.02,0.22] | [0.06,0.26] | [0.03,0.23] |
| Middle-low | 0.16^***^ | 0.15^***^ | 0.14^***^ | 0.13^**^ | 0.13^**^ |
|  | [0.08,0.24] | [0.07,0.23] | [0.06,0.22] | [0.05,0.21] | [0.05,0.21] |
| Middle | 0.14^***^ | 0.15^***^ | 0.15^***^ | 0.16^***^ | 0.16^***^ |
|  | [0.08,0.20] | [0.08,0.22] | [0.09,0.22] | [0.09,0.22] | [0.09,0.22] |
| Middle-high | -0.14^***^ | -0.13^***^ | -0.12^**^ | -0.12^**^ | -0.11^**^ |
|  | [-0.21,-0.06] | [-0.21,-0.06] | [-0.19,-0.05] | [-0.20,-0.05] | [-0.19,-0.04] |
| Highest | -0.31^***^ | -0.31^***^ | -0.30^***^ | -0.33^***^ | -0.31^***^ |
|  | [-0.39,-0.22] | [-0.40,-0.23] | [-0.38,-0.21] | [-0.42,-0.24] | [-0.39,-0.22] |
| Weight parameters |  |  |  |  |  |
| Origin | 0.37^***^ | 0.44^***^ | 0.45^***^ | 0.55^***^ | 0.53^***^ |
|  | [0.21,0.53] | [0.20,0.67] | [0.23,0.68] | [0.30,0.80] | [0.28,0.77] |
| Destination | 0.63^***^ | 0.56^***^ | 0.55^***^ | 0.45^***^ | 0.47^***^ |
|  | [0.47,0.79] | [0.33,0.80] | [0.32,0.77] | [0.20,0.70] | [0.23,0.72] |
| Social mobility |  |  |  |  |  |
| Upward | –––– | 0.03 | 0.02 | –––– | –––– |
|  | –––– | [-0.04,0.10] | [-0.05,0.09] | –––– | –––– |
| Downward | –––– | 0.07 | 0.06 | –––– | –––– |
|  | –––– | [-0.02,0.15] | [-0.02,0.15] | –––– | –––– |
| Short-range upward | –––– | –––– | –––– | 0.04 | 0.03 |
|  | –––– | –––– | –––– | [-0.04,0.12] | [-0.05,0.10] |
| Long-range upward | –––– | –––– | –––– | -0.02 | -0.02 |
|  | –––– | –––– | –––– | [-0.13,0.09] | [-0.12,0.09] |
| Short-range downward | –––– | –––– | –––– | 0.07 | 0.06 |
|  | –––– | –––– | –––– | [-0.02,0.16] | [-0.02,0.15] |
| Long-range downward | –––– | –––– | –––– | 0.15^*^ | 0.13 |
|  | –––– | –––– | –––– | [0.01,0.29] | [-0.01,0.26] |
| Socio-demographic controls |  |  |  |  |  |
| Age | 0.05^***^ | 0.05^***^ | 0.05^***^ | 0.05^***^ | 0.05^***^ |
|  | [0.03,0.06] | [0.03,0.06] | [0.03,0.06] | [0.03,0.06] | [0.03,0.06] |
| Male | 0.29^***^ | 0.29^***^ | 0.30^***^ | 0.29^***^ | 0.30^***^ |
|  | [0.24,0.35] | [0.24,0.35] | [0.24,0.35] | [0.23,0.34] | [0.24,0.35] |
| *Race/ethnicity (ref. white)* |  |  |  |  |  |
| Black | –––– | –––– | 0.18^***^ | –––– | 0.18^***^ |
|  | –––– | –––– | [0.11,0.24] | –––– | [0.11,0.24] |
| Hispanic | –––– | –––– | 0.06 | –––– | 0.07 |
|  | –––– | –––– | [-0.03,0.16] | –––– | [-0.03,0.16] |
| Other | –––– | –––– | 0.05 | –––– | 0.05 |
|  | –––– | –––– | [-0.09,0.19] | –––– | [-0.09,0.19] |
| Married (ref. unmarried) | –––– | –––– | -0.01 | –––– | -0.01 |
|  | –––– | –––– | [-0.07,0.05] | –––– | [-0.06,0.05] |
| Rural | –––– | –––– | 0.08^*^ | –––– | 0.07^*^ |
|  | –––– | –––– | [0.01,0.14] | –––– | [0.01,0.14] |
| AIC | 13753.85 | 13754.92 | 13289.56 | 13756.52 | 13291.95 |
| BIC | 13812.41 | 13826.50 | 13393.23 | 13841.11 | 13408.58 |
| Observations | 4713 | 4713 | 4713 | 4713 | 4713 |
