# Supplementary material for: Socioeconomic position, social mobility, and health selection effects on allostatic load in the United States
Source: PLoS One. 2021 Aug 4;16(8):e0254414. doi: 10.1371/journal.pone.0254414 (PMC8336836; doi:10.1371/journal.pone.0254414)
Supplement: S9 Table — Notes: * p < 0.05, ** p < 0.01, *** p < 0.001, 95% confidence intervals in parentheses. (DOCX) [file pone.0254414.s009.docx]

|  | Model 1 | Model 2 | Model 3 | Model 4 | Model 5 | Model 6 | Model 7 |
| --- | --- | --- | --- | --- | --- | --- | --- |
| *Immobile socioeconomic quintiles* |  |  |  |  |  |  |  |
| Low | 0.05 | 0.08 | 0.05 | 0.05 | 0.03 | 0.03 | 0.04 |
|  | [-0.05,0.16] | [-0.04,0.19] | [-0.06,0.15] | [-0.06,0.16] | [-0.07,0.14] | [-0.07,0.13] | [-0.07,0.14] |
| Middle | 0.10 | 0.08 | 0.10 | 0.11 | 0.12 | 0.12^*^ | 0.10 |
|  | [-0.00,0.20] | [-0.06,0.21] | [-0.02,0.21] | [-0.01,0.23] | [-0.00,0.24] | [0.01,0.23] | [-0.00,0.20] |
| High | -0.15^**^ | -0.15^**^ | -0.15^**^ | -0.16^**^ | -0.15^**^ | -0.15^**^ | -0.14^**^ |
|  | [-0.25,-0.06] | [-0.25,-0.05] | [-0.25,-0.05] | [-0.26,-0.06] | [-0.25,-0.05] | [-0.25,-0.06] | [-0.24,-0.03] |
| *Weight parameters* |  |  |  |  |  |  |  |
| Origin | 0.59^**^ | 0.96 | 0.88^*^ | 0.72^*^ | 0.77^**^ | 0.77^**^ | 0.68^*^ |
|  | [0.18,1.00] | [-0.20,2.11] | [0.16,1.61] | [0.14,1.29] | [0.23,1.31] | [0.30,1.25] | [0.16,1.19] |
|  |  |  |  |  |  |  |  |
| Destination | 0.41 | 0.04 | 0.12 | 0.28 | 0.23 | 0.23 | 0.32 |
|  | [-0.00,0.82] | [-1.11,1.20] | [-0.61,0.84] | [-0.29,0.86] | [-0.31,0.77] | [-0.25,0.70] | [-0.19,0.84] |
| *Social mobility* |  |  |  |  |  |  |  |
| Upward | –––– | -0.12 | -0.12 | –––– | –––– | –––– | –––– |
|  | –––– | [-0.33,0.09] | [-0.29,0.04] | –––– | –––– | –––– | –––– |
| Downward | –––– | 0.06 | 0.01 | –––– | –––– | –––– | –––– |
|  | –––– | [-0.16,0.28] | [-0.15,0.18] | –––– | –––– | –––– | –––– |
| Short-range upward | –––– | –––– | –––– | -0.12 | -0.14 | -0.14 | –––– |
|  | –––– | –––– | –––– | [-0.28,0.04] | [-0.30,0.02] | [-0.29,0.02] | –––– |
| Long-range upward | –––– | –––– | –––– | 0.04 | -0.01 | -0.01 | –––– |
|  | –––– | –––– | –––– | [-0.25,0.33] | [-0.30,0.27] | [-0.30,0.27] | –––– |
| Short-range downward | –––– | –––– | –––– | 0.04 | 0.01 | –––– | 0.04 |
|  | –––– | –––– | –––– | [-0.12,0.20] | [-0.15,0.16] | –––– | [-0.11,0.18] |
| Long-range downward | –––– | –––– | –––– | -0.04 | -0.04 | –––– | -0.03 |
|  | –––– | –––– | –––– | [-0.30,0.23] | [-0.29,0.22] | –––– | [-0.29,0.23] |
| *Socio-demographic controls* |  |  |  |  |  |  |  |
| Age | 0.04^**^ | 0.04^*^ | 0.04^*^ | 0.04^*^ | 0.04^*^ | 0.04^*^ | 0.04^**^ |
|  | [0.01,0.07] | [0.01,0.07] | [0.01,0.07] | [0.01,0.07] | [0.01,0.07] | [0.01,0.07] | [0.01,0.07] |
| Male | 0.31^***^ | 0.31^***^ | 0.34^***^ | 0.31^***^ | 0.34^***^ | 0.34^***^ | 0.34^***^ |
|  | [0.20,0.42] | [0.20,0.42] | [0.23,0.46] | [0.20,0.42] | [0.23,0.46] | [0.23,0.46] | [0.23,0.46] |
| *Race/ethnicity (ref. white)* |  |  |  |  |  |  |  |
| Black | –––– | –––– | 0.31^***^ | –––– | 0.31^***^ | 0.31^***^ | 0.31^***^ |
|  | –––– | –––– | [0.17,0.45] | –––– | [0.17,0.45] | [0.17,0.45] | [0.17,0.45] |
| Hispanic | –––– | –––– | 0.09 | –––– | 0.09 | 0.09 | 0.08 |
|  | –––– | –––– | [-0.08,0.27] | –––– | [-0.09,0.26] | [-0.09,0.26] | [-0.09,0.26] |
| Other | –––– | –––– | 0.00 | –––– | 0.00 | 0.00 | -0.01 |
|  | –––– | –––– | [-0.24,0.25] | –––– | [-0.24,0.24] | [-0.24,0.25] | [-0.25,0.23] |
| Married (ref. unmarried) | –––– | –––– | 0.02 | –––– | 0.02 | 0.03 | 0.01 |
|  | –––– | –––– | [-0.09,0.14] | –––– | [-0.09,0.14] | [-0.09,0.14] | [-0.10,0.13] |
| Rural | –––– | –––– | 0.19^**^ | –––– | 0.19^**^ | 0.19^**^ | 0.19^**^ |
|  | –––– | –––– | [0.07,0.31] | –––– | [0.07,0.31] | [0.07,0.31] | [0.07,0.31] |
| AIC | 4394.87 | 4396.74 | 4227.42 | 4399.38 | 4230.62 | 4226.71 | 4229.59 |
| BIC | 4431.95 | 4444.41 | 4301.16 | 4457.64 | 4314.90 | 4300.46 | 4303.33 |
| Observations | 1485 | 1485 | 1485 | 1485 | 1485 | 1485 | 1485 |
